# Oral Meloxicam Administration in Sows at Farrowing and Its Effects on Piglet Immunity Transfer and Growth

**DOI:** 10.3389/fvets.2021.574250

**Published:** 2021-02-11

**Authors:** Elena Navarro, Eva Mainau, Ricardo de Miguel, Déborah Temple, Marina Salas, Xavier Manteca

**Affiliations:** ^1^Department of Animal and Food Science, School of Veterinary Science, Universitat Autònoma de Barcelona, Bellaterra, Spain; ^2^Department of Animal Pathology, University of Zaragoza, Zaragoza, Spain

**Keywords:** pain, farrowing sow, piglet, immunity transfer, weight gain, meloxicam, immunoglobulin

## Abstract

Many factors can lead to an inadequate development of piglets during their first days of life, including poor maternal behavior, which can be due to pain caused by farrowing, and reduced colostrum ingestion. This study investigates the action of meloxicam administered orally at farrowing on piglet weight gain and immunity transfer. Thirty-five multiparous sows were divided into two groups and treated with 0.4 mg/kg of oral meloxicam (oral meloxicam group; *n* = 18) or with a mock administration (control group; *n* = 17). A total of 382 piglets were individually weighed on the farrowing day (day 0), as well as on days +9 and +20. Immunoglobulin G (IgG) and A (IgA) concentrations in piglet serum and in sow's saliva, colostrum and milk were measured. Additionally, Interleukin-2 (IL-2), Interleukin-4 (IL-4) and Interferon gamma (IFN-⋎) in serum of piglets and in sow's milk or colostrum were studied. All samples were obtained on days +1, +9, and +20. Piglets from sows in the oral meloxicam group tended to grow faster from day +9 to day +20 than did piglets from control sows (*p* = 0.059), and this difference was also observed in piglets with low body weight (BW) at birth (*p* = 0.056). The oral meloxicam group sows tended to increase the colostrum levels of IgA and IgG, as compared with control sows on day +1 (*p* = 0.068 and *p* = 0.072, respectively). IgA levels in piglet serum from the oral meloxicam group were significantly higher than in the control group on day +1 and +9 (*p* = 0.019 and *p* = 0.011 respectively). Furthermore, IL-2 and IL-4 levels in the serum of piglets from sows in the oral meloxicam group tended to be higher than that in the control group on day +9 (*p* = 0.078 and 0.056, respectively). The administration of meloxicam orally at the beginning of farrowing in multiparous sows increased immunoglobin and cytokine concentrations in colostrum, improving both humoral and cellular immune response of piglets. Pre-weaning growth of piglets born with a low BW improved in the meloxicam-treated group.

## Introduction

Piglets are born agammaglobulinemic because of the epitheliochorial placentation of swine (1, 2).

An early and sufficient intake of colostrum is crucial for piglet growth and survival (3, 4), as it is the source of energy as well as active and passive immunity. Colostrum is a complex mammary secretion released from the time of farrowing (early-colostrum) to 12 h (mid-colostrum) and up to 36 h post-farrowing (late-colostrum) (5). Early-colostrum is mostly produced before farrowing and contains up to 75% of Immunoglobulin G (IgG) and 20% of Immunoglobulin A (IgA), which are central elements of humoral immune responses. After farrowing, IgG concentration drastically drops, whereas IgA reduction during lactation is more gradual due to its role in the regulation of piglet intestinal microbiota, which is critical for the prevention of digestive problems (5).

Colostrum-associated cellular immunity has been overlooked for a long time. It contains around 10^6^ cells/mL, up to 25% of them being lymphocytes (6, 7). Immune responses are orchestrated via complex signaling pathways within cells mediated by cytokines, which are small proteins with a plethora of effects. IL-2, IL-4 and IFN-⋎ are important cytokine mediators of the adaptive immune response, thus their quantification allows for partial characterization of the immune response. IL-2 is mainly produced by T lymphocytes and induces the proliferation of T and B lymphocytes and the activation of Natural Killer cells (NK) (8–10). IL-4 triggers differentiation of T helper lymphocytes toward the Th2 subset, which is related to humoral and anthelmintic responses (8, 10), while IFN-⋎ activates macrophages and elicits the differentiation of T helper lymphocytes toward the Th1 subset, thus favoring cellular responses and boosting protection against intracellular microbes (10).

Non-steroidal anti-inflammatory drugs (NSAIDs) have analgesic, anti-inflammatory, anti-endotoxic and anti-pyretic effects. It has been proven that NSAIDs administered to sows help them recover from a painful situation such as lameness (11) or post-partum dysgalactia syndrome (12, 13). NSAIDs also decrease the mortality rate at weaning in litters from healthy sows (14) and in sows with dysgalactia syndrome (13). However, studies on the effect of NSAIDs, on sow welfare, piglet growth and immunity transfer in healthy sows show discrepancies. Meloxicam administered to healthy sows around farrowing improves post-farrowing sow recovery (15, 16) and enhances piglet growth, especially at weaning (17, 18) and in piglets with low body weight (BW) at birth (18, 19). Nevertheless, other studies administering NSAIDs to healthy sows around farrowing did not find enhanced sow welfare and recovery post-farrowing (20) or improve piglet growth (15, 21).

To our knowledge, only two studies have looked into the effects of NSAIDs administered to sows around farrowing and have assessed passive immunity transfer via colostrum and immune system development in piglets (18, 20). Both studies recorded IgG transfer measured in piglet serum without exploring immunoglobulins in sow colostrum or milk. These studies did not measure other relevant immune factors for piglet growth and survival, such as IgA or cytokines. Mainau et al. (18) demonstrated that the administration of meloxicam orally at the beginning of farrowing in multiparous sows increased the concentration of IgG in piglet serum and enhanced their pre-weaning growth.

The present study aims to evaluate the effect of meloxicam administered orally to healthy sows at the beginning of farrowing on piglet growth, also including the effect of sex and immune transfer via colostrum of immunoglobulins (A and G) and cytokines (Interleukins IL-2 and IL-4, and Interferon Gamma IFN-⋎), taking into account the sow parity effect.

## Materials and Methods

The experimental protocol described in this experiment was approved by the Institutional Animal Care and Use Committee of the Universitat Autònoma de Barcelona (CEEAH-1591) and the Generalitat de Catalunya (DMAH-6720). Written informed consent was obtained from the owners for the participation of their animals in this study.

### Animals, Housing and General Management

Sample size was calculated by means of ENE 3.0. The sow was the experimental unit. Based on two previous studies carried out by Mainau et al. (18, 19), a reference mean average daily gain (ADG) of 0.2 kg/day was established at sow level for the control group and an expected mean ADG of 0.225 kg/day was considered for the treatment group. An overall standard deviation of 0.025 kg/day (at sow level) was assumed with a power of 80% and confidence level of 95%. A prevision of 17 sows per group was predicted.

The experimental procedure was carried out on a commercial farm (Heura S.L.; Santa Perpètua de Mogoda, Barcelona, Spain), with 9 farrowing barns equipped with an evaporative cooling system each. From December 2017 to March 2018, a total of 35 hybrid (Landrace x Duroc) multiparous sows from 2nd to 7th parturition were randomly selected the day of farrowing. At least 5 replicates with 5 to 10 sows per replicate were studied.

On day 109 of gestation, sows were moved to the farrowing barn and were housed in individual farrowing crates (1.95 × 0.60 m) built with steel bars. Farrowing crates were centrally located in farrowing pens (2.40 × 1.80 m) with fully metal-slatted floors for sows and plastic-slatted floors for piglets. A metal pad ensured 36°C of heat for the piglets during the first week of life, and heat lamps were placed over the metal pad the first day of life. The temperature in the farrowing barn was kept constant at ~20°C, and the light was on from 07:00 to 17:00 h every day. Sows were offered 2.6 kg of feed per day, divided into two meals (07:00 and 15:00 h) and water was available *ad libitum* from drinkers.

Thirty days before farrowing, all sows were vaccinated with Clostridium novyi (2 mLSuiseng®, Hipra SA; Girona, Spain). Sixteen days after farrowing, sows that were not expected to be culled (*n* = 31 sows) were vaccinated with Parvovirus and Erysipelothrix rhusiopathiae (2 mL Eryseng®parvo, Hipra SA; Girona, Spain) and with Leptospira spp (2 mL Autovacuna®syva, Syva SAU; León, Spain). On day 113 of gestation, farrowing was hormonally induced with 2 mL of Planate^®^ (Cloprostenol 0,092 mg/mL, MSD Animal Health; Friesoythe, Germany) divided into two injections of 1 mL (07:00 h and 11:00 h). Only hormonally induced farrowings that started on the morning of day 114 of gestation were included in the study. Lame sows or those with any kind of visible disease symptoms such as mastitis, diarrhea, fever, or respiratory problems were not included in the study.

Treatments and manual interventions during farrowing followed the usual routine of the farm and were performed by the same person. The following treatments were allowed during farrowing and were administered intramuscularly (IM) in the neck. When the time interval between the birth of two piglets exceeded 1 h and the cervical canal was dilated, 1 mL of Oxytocin (Hormonipra®, HipraSA, Girona, Spain) was injected. When the cervical canal was not sufficiently dilated, sows were treated with 200 mg of Vetrabutine hydrochloride (Monzal®, Boehringer Ingelheim España; SA, Barcelona, Spain). When sows were very nervous around farrowing Carazolol (Suacron®, Divasa Farmavic SA; Barcelona, Spain) or Azaperone (Stressnil®, Janssen Animal Health, Elanco; Brussels, Belgium) were administered.

A total of 382 piglets, identified individually by a numeric ear tag, were included in the study. Piglets were weaned at 21 days of age, according to veterinary recommendations, and moved to another barn of the farm equipped with conditioned infrastructures for very young piglets.

### Experimental Procedure

In each replicate, sows were randomly allocated into two homogeneous groups, regarding parity, and treated with either 0.4 mg/kg body weight of meloxicam (Metacam® 15 mg/mL Oral Suspension, Boehringer Ingelheim Vetmedica GmbH; Ingelheim, Rhein, Germany) or a mock administration with an empty syringe. Treatments were administered at the beginning of the farrowing, between the first and the third piglet. If any further anti-inflammatory treatment was required, the sow was excluded from the study.

Litter size was standardized at 11–12 piglets by cross-fostering within 6–8 h post-farrowing. Cross-fostering was carried out within each treatment. Each treatment was identified with two different colored cards in order to make the treatment blind to farm and laboratory staff.

### Data Collection

For each sow, the following parameters were registered during farrowing by direct observations: the duration of farrowing (defined as the period of time between the first and the last piglet born), the condition of each piglet at birth (born alive, stillborn or mummified fetus), the piglet's sex (male or female), the number of treatments and manual interventions during farrowing, and the number of piglets cross-fostered and weaned. The presence of placenta retention was also recorded. During lactation, piglet mortality was registered. The number of sows culled after weaning and the interval between farrowing and the following fertile insemination were recorded.

One and 9 days after farrowing (day +1 and day +9) and the day before weaning (day +20), saliva samples were collected from all sows using Salivette® tubes (Sarstedt; Nümbrecht, Germany). Each tube contained a cotton swab, which was clipped with a Kocher clip, and sows were allowed to chew it for around 1 min. Then, the cotton swab was placed in the tube and centrifuged at 6,048 × g for 13 min. Saliva samples (~1–2 mL per cotton swab) were stored in Eppendorf tubes and frozen at −80°C until analysis. Colostrum and milk samples were collected from all sows on day +1 (colostrum) and on days +9 and +20 (milk). Sows were injected with 0.7 mLof Oxytocin IM (Hormonipra®, Hipra SA; Girona, Spain), and 30 s later, 2 mL of colostrum and milk were collected into sterile tubes. Colostrum and milk samples were immediately frozen at −20°C until analysis.

Each pig was individually weighed at farrowing (day 0), and on days +9 and +20. One day after farrowing, during one suckling event, 3–4 piglets of each litter were selected for blood sampling. Piglets were chosen so that at least one of them was suckling from the sow's craneal teats, another one from middle teats and yet another one from caudal teats. The same piglets from each litter were blood sampled on days +1, +9, and +20. Blood samples (1–2 mL) were collected into heparinized tubes from the anterior vena cava. Samples were centrifuged for 6 min at 2,058 × g and plasma was stored in Eppendorf tubes at −80°C until analysis.

All samples were analyzed at the Murcia University Veterinary Hospital Laboratory. Immunoglobulin G (IgG) and A (IgA) concentrations in piglet serum and sow saliva, colostrum and milk were quantified by using two commercially available sandwich ELISAs (IgA and IgG ELISA Quantitation Kit; Bethyl Laboratories; Montgomery, TX, USA). Interleukin-2 (IL-2), Interleukin-4 (IL-4) and Interferon gamma (IFN-⋎) in piglet serum, sow saliva, milk or colostrum were analyzed using MILLIPEX® MAP Porcine Cytokine/Chemokine Panel Kit (EMD Millipore; Darmstadt, Germany).

### Statistical Analysis

Data were analyzed using the SAS software (SAS Institute Inc.; Cary, NC, USA). The experimental unit for data analysis was the individual sow. All descriptive values in the Results section are shown as the mean ± standard error (SE). Significance was set at *p* < 0.05, and tendency at *p* < 0.1, in all cases.

The Mann-Whitney Wilcoxon test was used to test whether the performance values (other than piglet weight and average daily gain) obtained at the individual sow level were significantly different between treatments.

Normality tests of residuals were performed for each dependent variable. Weight of piglets and ADG (From birth to day+9, from day+9 to weaning and from birth to weaning) were normally distributed without data transformation. A general linear mixed model (proc MIXED) for repeated measures was used. Model included the fixed effects of treatment (control vs. oral meloxicam), day (at birth, day +9 and at weaning), sex (males vs. females) and their pair interactions. Day and piglet within sow were introduced as repeated effects. Weight at birth was introduced as a covariate for the analysis of weight at day +9 and at weaning, and litter size was introduced as a covariate in all the models. The residual maximum likelihood was used as a method of estimation. Differences in least-square means were investigated after using a Tukey adjustment for multiple comparisons. The same models were used to study the performance of piglets categorized by quintiles according to their weight at birth: very light (from 0.670 to 1.294 Kg), light (from >1.294 to 1.492 Kg), mid (from >1.492 to 1.625 Kg), heavy (from >1.625 to 1.878 Kg) and very heavy (from >1.878 to 2.427 Kg).

IgG and IgA concentrations in piglet serum, sow saliva and colostrum or milk, IL-2, IL-4 and IFN-⋎ in colostrum or milk, and IFN-⋎ in serum of piglets were normally distributed after a log transformation. IL-2 and IL-4 in piglet serum followed a normal distribution without data transformation. Additionally, extreme outliers detected by proc UNIVARIATE box plot procedures were deleted.

Immunity measurements in piglets and sows were analyzed using general linear mixed models (proc MIXED) for repeated measures. Models for immunity sow measurements included the fixed effects of treatment (oral meloxicam vs. control), day (day +1, +9, and +20), parity (from 2nd to 7th) and their pair interactions. Day was introduced as a repeated effect. Models for immunity piglet measurements included the fixed effects of treatment (oral meloxicam vs. control), day (day +1, +9, and +20), sex (male vs. female), the position at the udder (anterior, middle and posterior teats) and their pair interactions. Day and piglet within sow were introduced as repeated effects. The residual maximum likelihood was used as a method of estimation. Differences in least-square means were investigated after using a Tukey adjustment for multiple comparisons. All general linear mixed models included replicate (from 1 to 5) and farrowing barn (from 1 to 9) as random effects.

## Results

### Performance Parameters and Treatment Records

Results on performance and treatment records at the individual sow level are summarized in Table 1. Both treatment groups (oral meloxicam vs. control) were similar when the experimental procedure started in terms of performance data recorded during farrowing.

**Table 1:** Mean, standard error (SE), median (MED) and 95% confidence intervals for median (95% CI) of performance parameters and treatment records studied in the control and oral meloxicam groups during the whole trial period (from farrowing to weaning at 21 days).

	**Control** ** (*****n*** **=** **17 sows)**				**Oral meloxicam** ** (*****n*** **=** **18 sows)**				***P*-value**
**Items**	**Mean**	**SE^a^**	**MED**	**95%CI**	**Mean**	**SE**	**MED**	**95%CI**	
Parity	4.06	0.441	3	2–7	4.28	0.394	4	2–7	0.582
Piglets born at the moment of the treatment	1.70	0.143	2	1–3	1.78	0.117	2	1–3	0.856
Total duration of farrowing (h)	3.27	0.328	3.47	1.12–5.47	3.31	0.387	3.06	1.38–7.28	0.817
Total piglets born per litter	13.47	0.912	13	6–21	12.73	0.576	13	9–17	0.621
Live born per litter	11.88	0.624	12	6–18	11.36	0.584	11	8–16	0.489
Stillborn per litter	0.94	0.358	1	0–6	0.83	0.259	0.5	0–4	0.986
Mummified fetus per litter	0.65	0.226	0	0–2	0.55	0.217	0	0–3	0.815
Cross-fostered piglets per litter	11.18	0.231	11	10–13	10.67	0.256	10.5	8–12	0.209
Crushing deaths per litter	0.47	0.229	0	0–3	0.17	0.121	0	0–2	0.322
Total liveborn mortality	0.76	0.304	0	0–4	0.61	0.282	0	0–4	0.662
Total weaned piglets	10.41	0.193	10	9–12	10.06	0.338	10	6–12	0.569
Manual intervention per sow	0.47	0.174	0	0–2	0.38	0.230	0	0–4	0.422
Oxytocin treatment per sow	0.24	0.106	0	0–1	0.17	0.900	0	0–1	0.637
Total treatments per sow	0.24	0.106	0	0–1	0.33	0.140	0	0–2	0.731

*A total of 35 sows and 354 piglets were included in the study. P-value from Mann-Whitney Wilcoxon test is shown*.

a *SE, standard error*.

Treatment did not have an effect on the time interval between weaning and the following fertile insemination (4.769 ± 0.121 days in oral meloxicam group vs. 7.071 ± 1.811 days in the control group; *p* = 0.893), or on the number of sows culled after weaning (0.111 ± 0.076 in the oral meloxicam group vs. 0.176 ± 0.095 in the control group; *p* = 0.608).

Twenty-four piglets died during lactation, which represents 6.28% of mortality. Oral meloxicam treatment of sows did not significantly affect piglet mortality (6.84% from the control group and 5.73% from oral meloxicam group; *p* = 0.661).

The mean and standard error (SE) of weight at birth, weight on day +9 and weight at weaning are summarized in Table 2. Piglet weight at birth, on day +9 and at weaning was not different between the control and the oral meloxicam group. Piglet sex had a significant effect on the weight of the piglets, males being heavier than females at weaning (Table 1, Supplementary Material).

**Table 2:** Mean and standard error (SE) of the piglet weight at birth, 9 days after farrowing (day + 9) and at weaning (day + 20) in Kilograms and the Average Daily Gain (ADG) of piglets from birth to day + 9 after farrowing, from birth to weaning and from day + 9 to weaning in Kilograms per day for 354 piglets regarding treatment (control vs. oral meloxicam).

	**Control**		**Oral meloxicam**		
	**Mean**	**SE**	**Mean**	**SE**	***P*-value**
Weight at birth (Kg)	1.510	0.065	1.600	0.063	0.996
Weight at day +9 (Kg)	3.556	0.071	3.499	6.538	0.909
Weight at weaning (Kg)	6.479	0.109	6.538	0.103	0.644
ADG from birth to day +9 (Kg/day)	0.218	0.010	0.217	0.010	0.981
ADG from birth to weaning (Kg/day)	0.243	0.006	0.251	0.006	0.275
ADG from day +9 to weaning (Kg/day)	0.261^b^	0.007	0.275^a^	0.007	0.059

*Different superscripts (a, b) in the same row indicate significant differences within each effect (p < 0.05). Tendency has been shown at p < 0.1*.

Average daily gain (ADG) data are also summarized in Table 2. Oral meloxicam treatment of sows tended to increase piglet ADG from day +9 to weaning. Piglet sex had a significant effect on the ADG, males growing faster than females from birth to weaning and from day +9 to weaning.

Piglet weights (at birth, on day +9 and at weaning) and ADG (from birth to day +9, from day+9 to weaning and from birth to weaning) were not affected by treatment in piglets born with light, mid, heavy or very heavy weight at birth. Piglets with very light weight at birth tended to have a higher ADG from day+9 to weaning in the oral meloxicam group (267.93 ± 7.793 gr) than in the control group (240.11 ± 9.207 gr) (*p* = 0.056).

### Immunoglobulins G and A Concentrations in Saliva and Colostrum or Milk of Sows and in Piglet Serum

**Figure 1:**
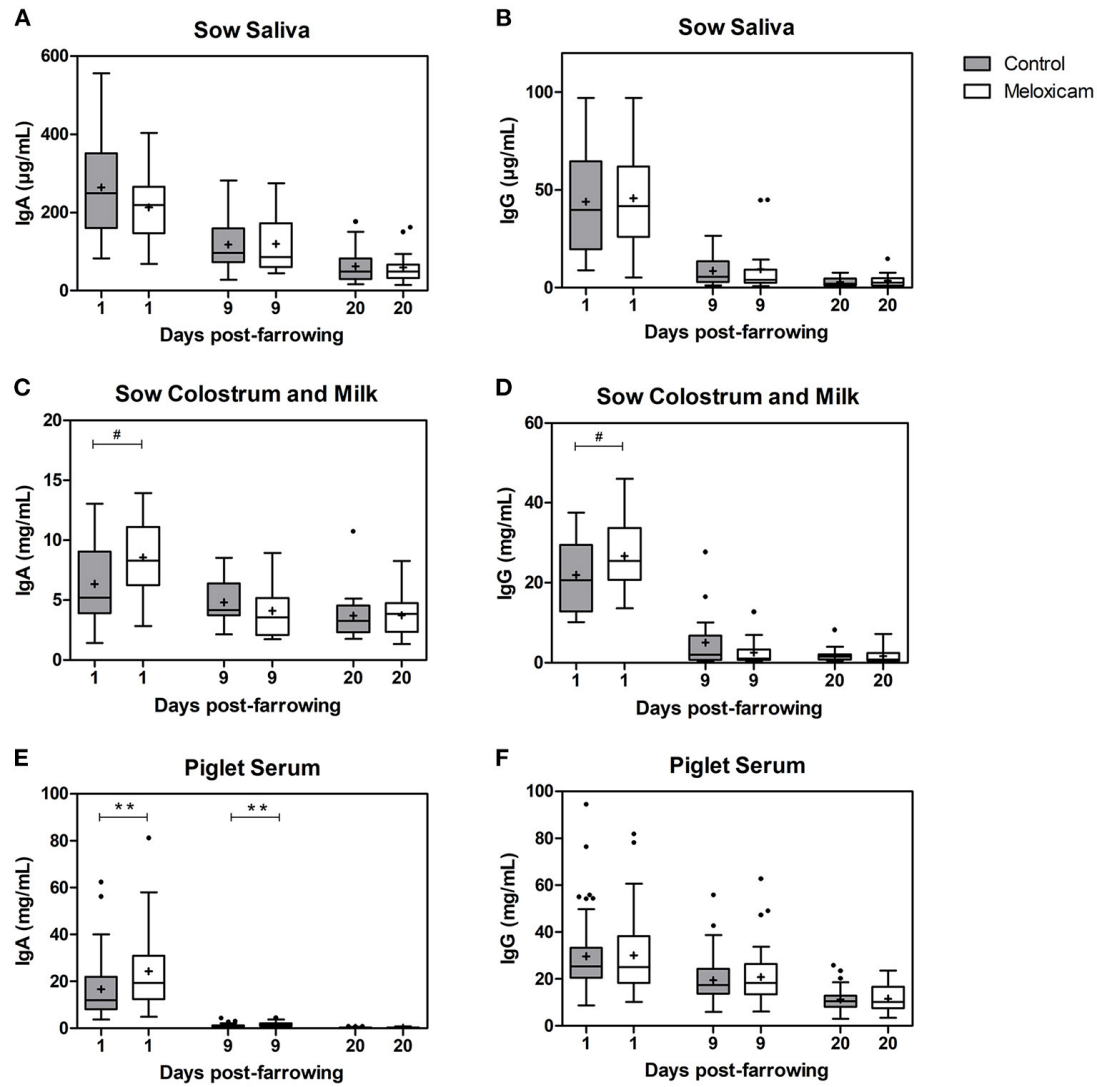
IgA and IgG at days +1, +9, and +20 after farrowing regarding treatment received by sows (control vs. oral meloxicam) in sow saliva **(A,B)**, sow colostrum and milk **(C,D)** and piglet serum **(E,F)**. Significant differences were established at *p* < 0.01(**) and tendency was set at *p* < 0.1(#). Boxes represent the interquartile range (IQ = Q3-Q1), horizontal lines inside the boxes represent the median and the cross (+) represents the mean values of the data. Whisker bars were calculated from the IQ (Upper: Q3 + 1.5 × IQ; lower: Q1 – 1.5 × IQ), and reflect the variability of the data outside Q1 and Q3. Points outside the box-and-whiskers plot represent extreme values of the population.

Immunoglobulin G and A (IgG and IgA) concentrations in saliva, colostrum or milk of sows and in piglet serum by treatment effect on days +1, +9, and +20 after farrowing are shown in Figure 1.

IgG levels in sow saliva (μg/mL) were affected by day after farrowing (day +1: 44.89 ± 4.486; day +9: 9.08 ± 1.810 and day +20: 3.28 ± 0.475; *p* < 0.001 in all pair comparisons), but were not affected by treatment (*p* = 0.547) irrespectively of the day studied.

IgA levels in sow saliva (μg/mL) were affected by day after farrowing (day +1: 239.64 ± 21.202, day +9: 118.477 ± 12.267 and day +20: 60.81 ± 7.295; *p* < 0.001 in all pair comparisons), but were not significantly affected by treatment; (*p* = 0.704) irrespective of the day studied.

IgG levels in colostrum or milk of sows (mg/mL) were affected by day after farrowing and were higher on day +1 (24.48 ± 1.484) than on days +9 (3.75 ± 0.953) and +20 (1.77 ± 0.298) (*p* < 0.001 in both cases). IgG levels in colostrum from the sows treated with oral meloxicam tended to be higher than in the control group on day +1 (*p* = 0.072). However, on days +9 and +20, IgG concentration in sow milk was not affected by treatment.

IgA levels in colostrum or milk of sows (mg/mL) were affected by day after farrowing and were higher on day +1 (7.48 ± 0.577) than on days +9 (4.41 ± 0.347; *p* < 0.001) and +20 (3.72 ± 0.315; *p* < 0.001). IgA concentration on day +9 and +20 were similar (*p* = 0.246). Furthermore, IgA levels in colostrum of sows treated with oral meloxicam tended to be higher than in the control group on day +1 (*p* = 0.068), but on day +9 and +20, IgA levels in sow milk were not affected by treatment.

IgA and IgG concentrations in saliva and in colostrum or milk were not affected by parity (saliva: *p* = 0.290 and *p* = 0.192, respectively, and colostrum or milk: *p* = 0.127 and *p* = 0.232). The interaction between treatment and parity was not significant (saliva IgA *p* = 0.113; IgG *p* = 0.925 and colostrum or milk IgA *p* = 0.239; IgG *p* = 0.112).

IgG levels in piglet serum (mg/mL) were affected by day after farrowing (day +1: 29.93 ± 1.377; day +9: 20.26 ± 0.935 and day +20: 11.48 ± 0.466; *p* < 0.001 in all pair comparisons). IgG levels in piglet serum were not significantly affected by treatment (*p* = 0.963), sex (*p* = 0.189) or piglet position at the udder (*p* = 0.811) irrespective of the day studied.

IgA levels in piglet serum (mg/mL) were affected by day after farrowing (day +1: 20.63 ± 1.314, day +9: 1.36 ± 0.080 and day +20: 0.27 ± 0.018 *p* < 0.001 in all pair comparisons), and there was an interaction between treatment and sampling day (*p* = 0.020). IgA levels in piglet serum from sows treated with oral meloxicam were significantly higher than in piglets from the control group on day +1 (*p* = 0.019) and day +9 (*p* = 0.011). However, on day +20, IgA level in piglet serum was not significantly affected by treatment (*p* = 0.943). IgA levels in piglet serum were not significantly affected by sex (7.79 ± 0.944 in females vs. 7.89 ± 1.001 in males; *p* = 0.633) or by piglet position at the udder (anterior teats: 7.67 ± 1.048, middle teats: 7.17 ± 1.328 and posterior teats: 9.07 ± 1.448, *p* = 0.725) irrespective of the day studied.

### Concentration of Cytokines (IL-2, IL-4 and IFN-**⋎**) in Colostrum or Milk of Sows and in Piglet Serum

**Figure 2:**
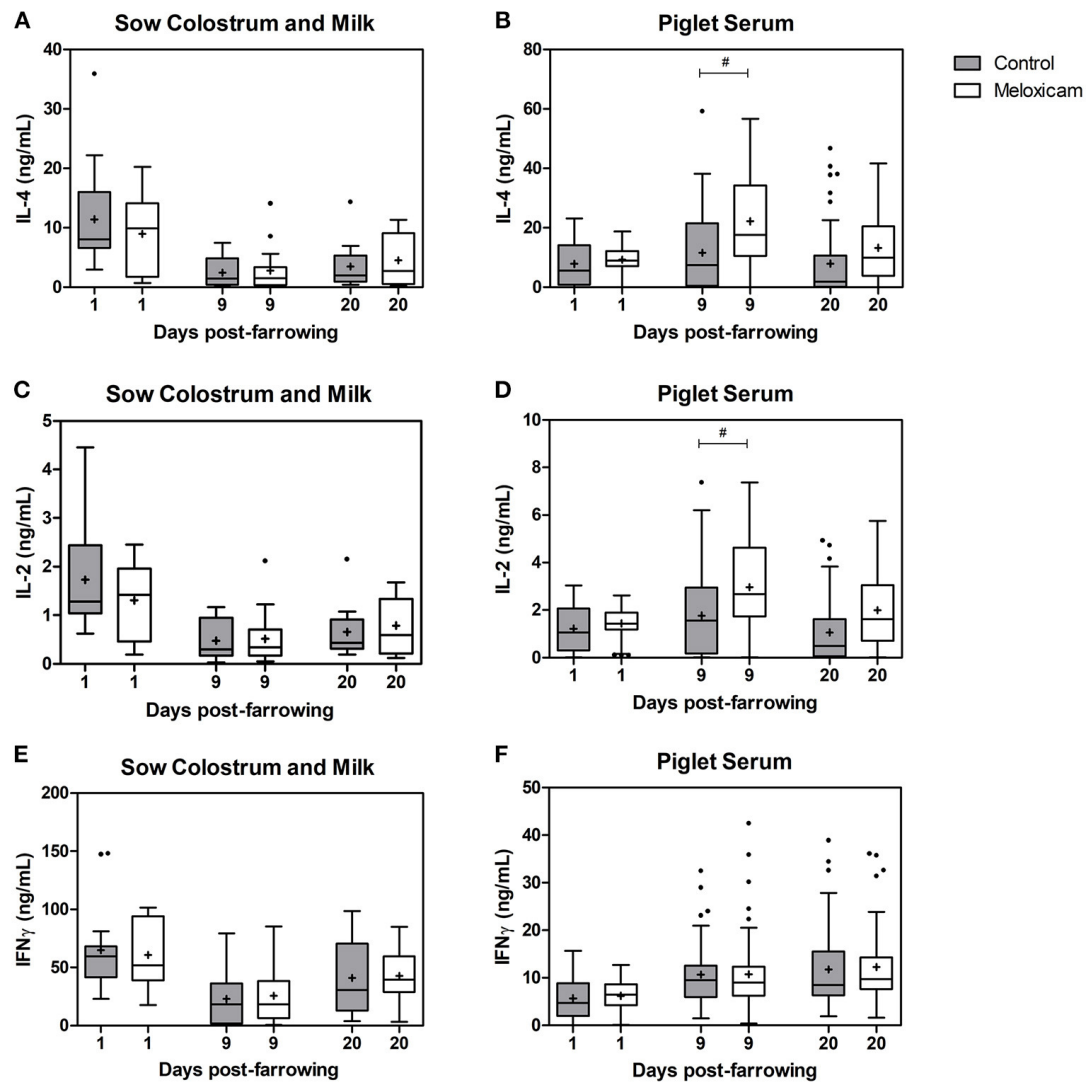
Concentration of IL-4, IL-2, and IFN-⋎ at days +1, +9, and +20 after farrowing regarding treatment received by sows (control vs. oral meloxicam) in sow colostrum and milk (a, c, e) and piglet serum (b, d, f). Tendency differences were established at *p* < 0.1(#). Boxes represent the interquartile range (IQ = Q3 – Q1), horizontal lines inside the boxes represent the median and the cross (+) represents the mean values of the data. Whisker bars were calculated from the IQ (Upper: Q3 + 1.5 × IQ; lower: Q1 - 1.5 × IQ) and reflect the variability of the data outside Q1 and Q3. Points outside the box-and-whiskers plot represent extreme values of the population.

Concentration of interleukins (IL-2 and IL-4) and interferon gamma (IFN-⋎) in colostrum or milk of sows and in piglet serum by treatment effect on days +1, +9, and +20 after farrowing are shown in Figure 2.

IL-2, IL-4 and IFN-⋎ concentration in colostrum or milk of sows (ng/mL) were affected by day after farrowing (*p* < 0.001 in all cases). IL-2, IL-4 and IFN-⋎ in sow colostrum on day +1 (IL-2: 1.51 ± 0.166; IL-4: 10.12 ± 1.289; IFN-⋎: 62.99 ± 5.505) showed higher concentrations than in sow milk on day +9 (IL-2: 0.50 ± 0.079; IL-4: 2.60 ± 0.540; IFN-⋎: 24.31 ± 3.827) and on day +20 (IL-2: 0.73 ± 0.089; IL-4: 4.02 ± 0.641; IFN-⋎: 41.86 ± 4.585), whereas concentrations on day +20 were higher than on day +9.

IL-2, IL-4, and IFN-⋎ concentrations in colostrum or milk of sows were not significantly affected by treatment (*p* = 0.206, 0.142, and 0.322 respectively).

IL-2, IL-4 and IFN-⋎ concentrations in colostrum or milk of sows were affected by parity (*p* = 0.010, *p* < 0.001 and *p* = 0.008 respectively). The general pattern was that sows in their second parity showed lower levels of cytokines than did older sows (three parturitions or more). Specifically, IL-2 levels in colostrum or milk of sows in their second parity were lower than those in sows in their fourth (*p* = 0.016) and fifth parity (*p* = 0.023) and tended to be lower than in sows in their seventh parity (*p* = 0.098). IL-4 levels in colostrum or milk of sows in their second parity were lower than those in sows in their third (*p* = 0.005), fourth (*p* = 0.001), fifth (*p* = 0.001), sixth (*p* = 0.035) and seventh parity (*p* = 0.002). IFN-⋎ levels in colostrum or milk of sows in their second parity were lower than those in sows in their fourth parity (*p* = 0.008) and tended to be lower than in sows in their sixth parity (*p* = 0.070).

IL-2 and IL-4 levels in piglet serum (ng/mL) were affected by day after farrowing, and were higher on day +9 (IL-2: 2.40 ± 0.173; IL-4: 17.15 ± 1.442) than those on days +1 (IL-2: 1.33 ± 0.070; IL-4: 8.53 ± 0.526; *p* < 0.001 in both cases) and +20 (IL-2: 1.55 ± 0.145; IL-4: 10.67 ± 1.156; *p* < 0.001 and *p* = 0.001, respectively).

IL-2 and IL-4 levels in piglet serum were affected by treatment, and tended to be higher in the oral meloxicam group than those in the control group on day +9 (*p* = 0.078 and *p* = 0.056, respectively). IL-2 and IL-4 levels in piglet serum were not significantly affected by sex of piglets (*p* = 0.596 and *p* = 0.868, respectively) or by piglet position at the udder (*p* = 0.888 and *p* = 0.715, respectively) irrespective of the day studied.

IFN-⋎ levels in piglet serum (ng/mL) were affected by day after farrowing, and were lower on day +1 (6.00 ± 0.334) than those on day +9 (10.74 ± 0.654) and day +20 (12.07 ± 0.758) (*p* < 0.001 in all cases).

IFN-⋎ levels in piglet serum were not significantly affected by treatment (*p* = 0.409), sex of piglets (*p* = 0.858), or piglet position at the udder (*p* = 0.320), irrespective of the day studied.

## Discussion

In the present study, both treatment groups (oral meloxicam and control) were well-matched in terms of performance variables recorded during farrowing. Early administration of oral meloxicam treatment did not negatively affect total piglets born alive per litter, total duration of farrowing, treatments administered during farrowing (such as oxytocin) or the number of manual interventions during farrowing. Hence, it appears that the use of oral meloxicam during parturition (more specifically between the first and the third piglet born) did not interfere with the progression of the birth process.

### Piglet Mortality and Growth

In agreement with other authors that studied the effect of NSAIDs around farrowing (17, 18, 20, 21), oral meloxicam administered to healthy sows did not show an effect on pre-weaning piglet mortality. On the contrary, Homedes et al. (14), in a large-scale study on commercial farms with a high incidence of pre-weaning mortality (±10%), showed lower pre-weaning piglet mortality after ketoprofen administration to sows within 12 h after farrowing. Homedes et al. (14) explained such an effect due to higher milk production by the sow ketoprofen-treatment group. We assume that a larger sample size enrolling different farms with high pre-weaning mortality would be needed to observe differences in piglet mortality (piglet mortality in our study was 6.8%).

Piglet weights at birth were similar (16, 17) or slightly higher (3, 22, 23) than values reported in other studies. The administration of oral meloxicam at the beginning of farrowing tended to enhance the ADG of piglets from day +9 to weaning, and particularly for the lightest piglets. A similar effect was described by Mainau et al., in two studies (18, 19), treating the sows around farrowing with injectable and with oral meloxicam. Tenbergen et al. (17), injected meloxicam intra-muscularly within 12 h of farrowing and found that piglet ADG tended to be higher for piglets from the meloxicam group sows than for control piglets in medium-sized litters (11–13 piglets). Ketoprofen is another AINE used in pig production, but Viitasaari et al. (21) and Ison et al. (15), who both injected sows with ketoprofen during farrowing, did not find that it had any effect on piglet average daily gain to weaning. These discrepancies in the effects of NSAIDs administered to healthy sows around farrowing on piglet growth could be due to different factors such as the active principle administered. Meloxicam is a selective COX-2 inhibitor and may be a more specific treatment for inflammation caused by farrowing than a non-selective COX inhibitor, like ketoprofen (24). The time of administration is another important factor to take into consideration. Studies administering meloxicam at the beginning of farrowing (17–19) show the effect on weaning weights of piglets and ADG. Thus, the active ingredient administered (preferably a selective COX-2 inhibitor) and the administration time (as soon as possible after farrowing starts) are presumably important factors to improve piglet growth and weight at weaning.

### Transfer of Passive and Active Immunity

Colostrum intake is crucial for development of piglet immunity. In this study, and in accordance with normal colostrum and milk immunoglobulin kinetics (5), sow colostrum and milk IgG and IgA showed an abrupt and steady decrease respectively (Figures 1C,D). Interestingly, colostrum immunoglobulin content on day +1 was higher in the oral meloxicam group then in the control group. The difference between groups was more pronounced in IgA than in IgG, which could be explained by the switch between the IgG/IgA ratio after farrowing (5). One-hundred percent of colostrum IgG and 40% of colostrum IgA come from sow blood via an Ig receptor, whereas up to 60% of IgA is directly synthesized in the mammary gland (1). Our data support the local role of oral meloxicam in the mammary gland, which likely decreases local inflammation, thus favoring both immunoglobulin recruitment from plasma and local production of IgA in plasma cells (1). Indeed, *in vitro* studies developed in cattle have shown the anti-inflammatory effect of meloxicam in mammary epithelial cells (25). Furthermore, mastitis in cows has been associated with reduced pre-weaning immunity, growth, and health of the offspring (26, 27), so the anti-inflammatory effect elicited by meloxicam treatment is presumed to have the opposite effect.

In comparison with blood sampling, saliva sampling is generally considered to be a non-invasive and stress-free methodology (28). IgG levels in sow saliva are directly proportional to the levels in sow serum, whereas IgA in saliva is mostly produced locally, so IgA levels are highly variable in response to environmental factors such as stress and oral infections (29). In our study, saliva IgG levels, a marker of plasma IgG levels, showed no differences between groups, which probably rules out a systemic effect of oral meloxicam administration on the Ig increase in piglets from treated sows.

IgA and IgG concentration in piglet serum during lactation is the result of intake of immunoglobulins from colostrum. The quick drop of IgA and the slow drop of IgG in piglet serum is likely explained by the different half-lives of these immunoglobulins in serum, being 6 days for IgA and 21 days for IgG (30). IgA concentration was higher in piglet serum in the oral meloxicam group on days +1 and +9. Interestingly, diarrhea of newborn piglets is one of the biggest health issues in pig production, and increased IgA levels could play a major role in preventing these problems by their protective effect on the intestinal mucosa (1). Mainau et al. (18) found that the administration of meloxicam orally at the beginning of farrowing (on average, when 2.6 piglets had already been born) increased the concentration of IgG in the serum of piglets. In the present study, sows were treated early at the beginning of farrowing, when early colostrum (with the highest IgG levels) has already been produced and thus the influence of treatment on the IgG serum levels of piglets fed with this colostrum was lower. Nevertheless, weaker piglets and those born later during parturition are known to suffer from delayed and reduced colostrum intake (31). These animals have lower survival and growth rates, which may be improved by treatment with meloxicam, as shown by our results with piglets born with a very light weight at birth, as well as by other studies (31). These differences could be explained by a higher IgG and IgA immunity transfer in the treatment group in these weaker animals, which are likely to consume a smaller quantity of early-colostrum and a larger proportion of mid- and late-colostrum. Unfortunately, in this study a low percentage of piglets with low BW at birth were blood sampled, thus hampering a proper analysis of their serum IgG and IgA levels.

Regarding colostrum and milk cytokines, higher levels of IL-2, IL-4, and IFN-Y were found on day +1, which is likely to be related to pain and to contamination of the reproductive tract induced by farrowing. Milk cytokine levels moderately decreased between day +1 and +9 and increased again between day +9 and +20, likely in response to the vaccination given to sows on day +16. Cytokines and lymphoid cells have been demonstrated to cross the intestinal barrier of newborn piglets (32–34). In piglet serum, cytokine levels measured on day +1 are expected to be the result of both colostrum-derived cytokines and cytokines produced by the piglets. In contrast, taking into consideration the short half-life of these cytokines (minutes for IL-2 and IL-4 and a few hours for IFN-⋎) and the loss of piglet intestinal permeability, cytokine levels on days +9 and +20 reflect only the activity of the piglet immune system. Higher concentrations of all cytokines were found on day +9, likely due to the immune challenge elicited by tail docking (in both sexes) and castration (in males), which were performed on their second day of life. Interestingly, higher IL-4 and IL-2 levels were measured in piglets from the meloxicam treated group on days day +9 (Figures 2B,D). Increased secretion of IL-4 and IL-2 in piglets has been related to better Th2 and Th1 immune responses, respectively (8). Moreover, IL-4 induces antibody production and tissue repair, whereas IL-2 plays a major role in the activation of NK-cells and the generation of effector and memory cells (9, 10). This positive influence of colostrum on the immune system development could be related to the transfer of colostrum-associated immune cells, which are absorbed selectively in the newborn gut, although the precise mechanisms remain unclear (1). Therefore, it could be hypothesized that meloxicam treatment around farrowing had an impact on the concentration of immune cells in colostrum, but further studies are needed to investigate this hypothesis.

This study was developed on a commercial farm with really good sanitary and husbandry conditions. Further research is required to determine if these positive results on piglet welfare can be even more pronounced by studing a larger set of commercial farms with higher mortality rates and lower growth rates during lactation. In summary, the results of this study show that early administration of oral meloxicam improves some aspects of piglet performance and welfare. Further research is needed to study whether these effects are also observed in primiparous sows or could be improved by administering meloxicam before the onset of farrowing.

## Conclusions

The administration of meloxicam orally at the beginning of farrowing in multiparous sows increased the concentration of immunoglobins and cytokines in sow colostrum and improved both humoral and cellular immune response in piglets. Pre-weaning growth of piglets, especially in piglets born with low BW, tended to be higher in the meloxicam-treated group than that in the control group.

## Data Availability Statement

The original contributions presented in the study are included in the article/Supplementary Material, further inquiries can be directed to the corresponding author/s.

## Ethics Statement

The experimental protocol described in this experiment was approved by the Institutional Animal Care and Use Committee of the Universitat Autònoma de Barcelona (CEEAH-1591) and the Generalitat de Catalunya (DMAH-6720). Written informed consent was obtained from the owners for the participation of their animals in this study.

## Author Contributions

EN: data acquisition, structuralization, and interpretation, drafting of the manuscript and final approval of the version to be published. EM: study concept and design, data acquisition, data analysis and interpretation, drafting of the manuscript and final approval of the version to be published. RM: immunology data analysis and interpretation, preparation of figures, and drafting of the manuscript and final approval of the version to be published. DT and MS: data acquisition and final approval of the version to be published. XM: study concept and design, drafting of the manuscript and final approval of the version to be published. All authors contributed to the article and approved the submitted version.

## Conflict of Interest

The authors declare that this study received funding from Boehringer Ingelheim Vetmedica GmbH. The funder was not involved in the study design, collection, analysis, interpretation of data and the writing of this article.
