# Supplementary material for: Oral Meloxicam Administration in Sows at Farrowing and Its Effects on Piglet Immunity Transfer and Growth
Source: Front Vet Sci. 2021 Feb 11;8:574250. doi: 10.3389/fvets.2021.574250 (PMC7928392; doi:10.3389/fvets.2021.574250)
Supplement: Supplementary file 1 [file Table_1.DOCX]

Supplementary Material

# Supplementary Tables

**Table 1.** Mean and standard error (SE) of the piglet weight at birth, 9 days after farrowing (day+9) and at weaning (day +20) in Kilograms and the Average Daily Gain (ADG) of piglets from birth to day+9 after farrowing, from birth to weaning and from day+9 to weaning in grams per day for 354 piglets regarding piglet sex (females vs. males) effects.

|  | **Male** | | **Female** | |  |
| --- | --- | --- | --- | --- | --- |
|  | **Mean** | **SE** | **Mean** | **SE** | **P-value** |
| **Weight at birth (Kg)** | 1.583 | 0.046 | 1.527 | 0.047 | 0.566 |
| **Weight at day +9 (Kg)** | 3.533 | 0.061 | 3.523 | 0.063 | 0.944 |
| **Weight at weaning (Kg)** | 6.601^a^ | 0.099 | 6.415^b^ | 0.103 | 0.016 |
| **ADG from birth to day +9 (Kg/day)** | 0.220 | 0.008 | 0.215 | 0.009 | 0.388 |
| **ADG from birth to weaning (Kg/day)** | 0.253^a^ | 0.005 | 0.241^b^ | 0.006 | 0.024 |
| **ADG from day +9 to weaning (Kg/day)** | 0.277^a^ | 0.007 | 0.260^b^ | 0.007 | 0.005 |

*Different superscripts (a, b) in the same column indicate significant differences within each effect (p < 0.05). Tendency has been shown at p < 0.1.*
